# A Computational Approach towards Visual Object Recognition at Taxonomic Levels of Concepts

**DOI:** 10.1155/2015/905421

**Published:** 2015-06-22

**Authors:** Zahra Sadeghi, Babak Nadjar Araabi, Majid Nili Ahmadabadi

**Affiliations:** ^1^Cognitive Robotics Lab, School of Electrical and Computer Engineering, University of Tehran, Tehran 14395-515, Iran; ^2^School of Cognitive Sciences, Institute for Research in Fundamental Sciences (IPM), Tehran 19395-5746, Iran

## Abstract

It has been argued that concepts can be perceived at three main levels of abstraction. Generally, in a recognition system, object categories can be viewed at three levels of taxonomic hierarchy which are known as superordinate, basic, and subordinate levels. For instance, “horse” is a member of subordinate level which belongs to basic level of “animal” and superordinate level of “natural objects.” Our purpose in this study is to take an investigation into visual features at each taxonomic level. We first present a recognition tree which is more general in terms of inclusiveness with respect to visual representation of objects. Then we focus on visual feature definition, that is, how objects from the same conceptual category can be visually represented at each taxonomic level. For the first level we define global features based on frequency patterns to illustrate visual distinctions among artificial and natural. In contrast, our approach for the second level is based on shape descriptors which are defined by recruiting moment based representation. Finally, we show how conceptual knowledge can be utilized for visual feature definition in order to enhance recognition of subordinate categories.

## 1. Introduction

Categorization is the first step in recognition and so it is fundamental for perception, communication, and any kind of interaction with the environment [1]. The goal of categorization is to partition the search space into different groups in such a way that members of each group reflect a similar concept or idea. According to research in conceptual developments, there exist different strategies for object categorization. Three different types of categorization are identified by cognitive science researchers based on whether the similarities are defined by their external relations or internal properties. These are known as thematic categorization, script categorization, and taxonomic categorization. Thematic categorization focuses on the spatially or contiguous relationship between objects. For example, dog and leash are in the same thematic category. In script categorization objects with similar roles or functionality are grouped together. For instance, egg and cereal belong to the same script category. Finally, taxonomic categorization refers to a hierarchy which is constructed in an ascending order of inclusiveness (e.g., like terrier-mammal-animal) [2]. While the members of the first two types of categorization do not necessarily share similar properties, in the taxonomic categorization, objects are grouped based on similar observable features. Thereupon, taxonomic organization is applicable to visual object categorization in which object appearance plays a determinant role in object recognition and classification.

This paper is organized as follows. We first explain three levels of concepts in taxonomic categorization. Then, in Sections 3 and 4, computational models are presented for feature definition at the first level (i.e., superordinate level) and second level (i.e., basic level) of taxonomy. Finally, in Section 5, we show that conceptual object representation improves accuracy of subordinate categorization.

## 2. Three Taxonomic Levels of Concepts

It has been well demonstrated that infants, children, and adults use different levels of inclusiveness for object naming and categorization [3–5]. This semantic taxonomical model known as superordinate-basic-subordinate categorization is constituted of three levels of abstraction and is shown in Table 1 [6–8]. The degree of inclusiveness is highest at the top level and decreases approaching the bottom of the tree. It is not clearly known which of these levels is primarily used in recognition of objects and it is generally accepted that identification of each level relies on several parameters like the object familiarity and frequency as well as the context in which the object is viewed. Also, the number of hierarchical levels is variable among different groups of people according to their level of expertise. Rosch et al. have shown that people use the basic level as a preferred class for recognition of objects. They posit that this level constitutes optimal information for quick categorization [8]. In contrast to this claim, other studies have challenged this idea by showing that human perceive superordinate distinction prior to basic level and it occurs at early stages of processing visual information [9–11]. One supporting explanation behind top-down design is declared to be survival reasons because coarse information obtained from quick processing will promote an immediate appropriate reaction [12].

**Figure 1:**
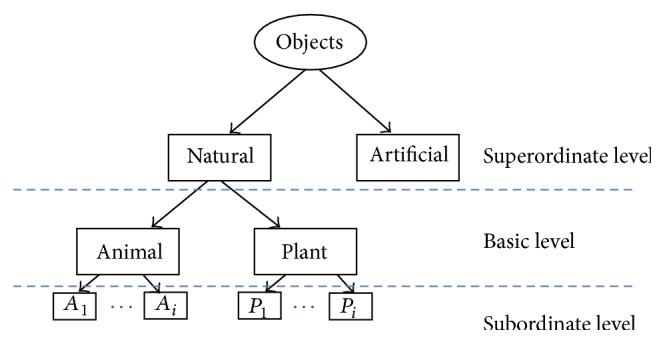
Taxonomic structure of recognition used in this paper. *A*_*i*_ and *P*_*i*_ refer to the subcategories of animal and plant correspondingly.

**Figure 2:**
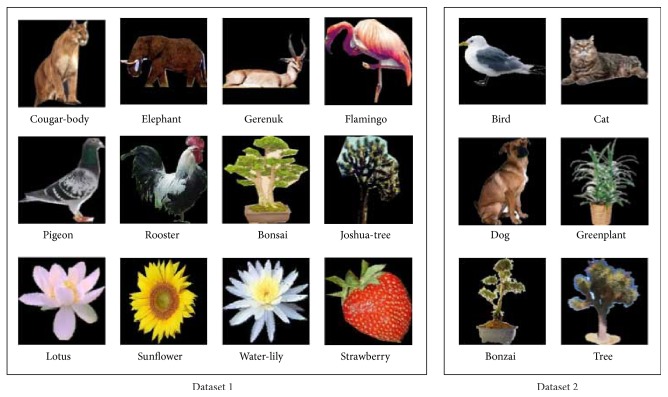
Sample of animal and plant subcategories.

In this paper, we consider a more general taxonomic structure with an onset on artificial versus natural groups in the very first step of bifurcation of all items. This is illustrated in Figure 1. This structure is adopted according to the visual properties of objects. It has been previously shown by Oliva and Torralba that scene images can be semantically discriminated along artificial to natural axis at the superordinate level of categorization [13]. The natural supercategory might then be subdivided into animal and plant subcategories at the basic level. Thereupon, our terminology for superordinate, basic, and subordinate categories is slightly different from what has been broadly used in the literature of psychology. For instance, we assume that “horse” is located in subordinate level which belongs to the basic level of “animal” and the superordinate level of “natural” objects. Our investigation for the tree structure is only devoted to natural objects behind which there's a stronger theory of hierarchical semantic structure. For instance, according to folk biology, regardless of their culture, people have a similar taxonomic structure for thinking about living subcategories as animals and plants [14]. There are also a number of studies advocating the superiority of tree-structure for capturing taxonomic relationships among biological data [15, 16]. Following this structure, for a classification task, each image is associated with three different labels corresponding to each particular level of inclusiveness. In the following sections we describe our computational approach for feature definition at each level of concept. For evaluation, we collected benchmark data from Caltech-101 and coil-100 image databases, MPEG-7, and the stimuli database gathered by Konkle et al. [17]. More details about the categories and their labels are shown in Table 2. It should be mentioned that we created conceptual categories of animal and plant based on classes available in each database. For plant categories, we could only find 6 such classes in Caltech 101. Hence, in order to place an equal chance level of 1/6 in both subcategories of animals and plants, we only selected 6 subclasses for the animal class as well. The subclasses are selected such that three of them are quadruped animals and the other three are birds. Note that all objects are segmented before the whole process of recognition using annotation information associated to each object class. The objects are then cropped to reduce the area of background of images. The stimuli database provided by Konkle et al. contains colorful images of isolated objects with a plain background. In contrast, images from MPEG-7 are isolated objects in binary format and hence they are only used in Section 4 for basic level categorization. One example of each natural subcategory for the first two datasets is shown in Figure 2.

## 3. Superordinate Level of Recognition

The first level of inclusiveness in hierarchy of concepts consists of two supplementary groups of items, that is, artificial and natural entities. Intrinsically, all objects can be considered as belonging to one of the conceptual categories of either artificial or natural items based on their inherent source of creation. In other words, objects can be classified as either human-made (artificial) or non-human-made (natural) entities. Breaking up all existent items in such a way can be contemplated as the utmost general course of viewing the world; that is, objects are assumed to be made by mankind or they are found in the nature without human interference. In addition, we are inclined to think that distinguishing objects at the first level of taxonomy is independent of prior knowledge and that this distinction can be made in an unsupervised manner. This is in accord with the top-down model proposed by Bar in which coarse information derived from a visual input directly activates similar high level representations without making an exhaustive search to find a similar stored representation in memory [12].

**Figure 3:**
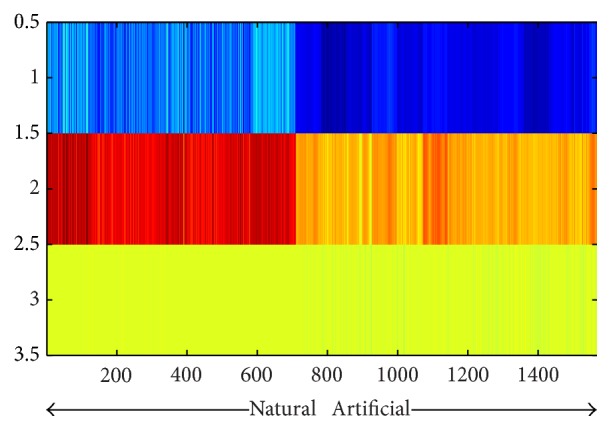
Frequency features for all data. In an up-down direction, vertical axis represents the three dimensions defined in (4) to (6).

The contributing role of semantic content in making a broad distinction of images has been studied on scene images [13] as well as isolated object categories [18] and frequency-based features have indicated efficient results in capturing the superordinate characteristics of objects. Specifically, the frequency attributes of objects are defined using the following equations [18]:

(1) FI=Finput_image,

(2) magnitudex,y=Re⁡FIx,y2+Im⁡FIx,y2,

(3) phasex,y=tan−1⁡ImFIx,yRe⁡FIx,y,

(4) FreqFeat1=∑x,y∈input_imagemagnitudex,y,

(5) FreqFeat2=∑x,y∈input_imagelog⁡1+magnitudex,y,

(6) FreqFeat3=∑x,y∈input_imagephasex,y,

where FI is the result of Fourier transform of gray scale input image.  Figure 3 illustrates the distinguishable values captured by the visual features explained via (4) to (6) using dataset 1. It can be seen that the three dimensions are all containing distinctive values for grouping objects in two separate groups. In addition, we performed a clustering task on the obtained feature values to evaluate the discrimination characteristics of the feature sets in an unsupervised manner. The results are evaluated by using *f*-measure, precision, recall, and accuracy and are compared with Gabor [19] and C2 features [20] in Table 3. In the appendix we provide further analytical figures (Figures 7, 8, and 9) which indicate the potent discrimination obtained by the defined feature sets.

## 4. Basic Level of Recognition

In this section, we address the problem of basic category representation. This is the second level in the taxonomic structure which is associated with the general classes within natural superordinate category. The purpose of this phase is to investigate the visual distinction between animal and plant classes. Hence, we deal with two broad semantic subcategories of natural objects (i.e., animals and plants). A tremendous amount of research has been conducted on object recognition based on local properties of objects (HOG [21], C2 [22], SIFT [23], and LBP [24]). In contrast, in our approach, in order to distinguish between the conceptual categories at the basic level, we utilize shape descriptors to extract global main discriminations between animal and plant categories. The theory behind this approach is that the categories of animal and plant are distinguishable in form and configuration, and hence using global features by applying shape descriptors can be profitable.

### 4.1. Method

**Figure 4:**
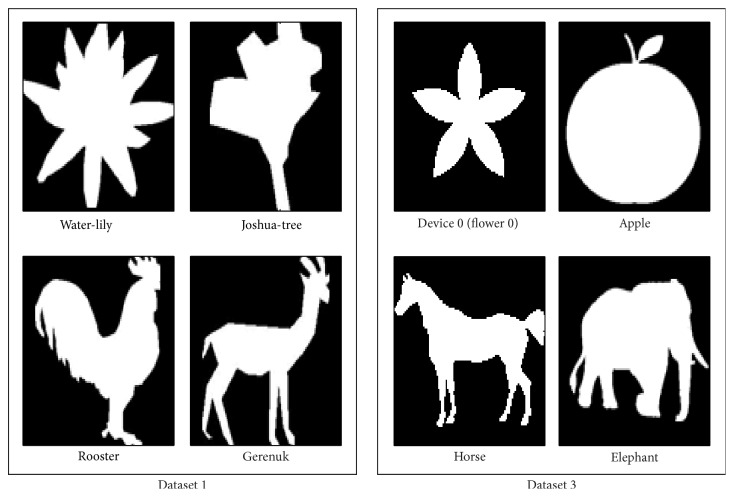
Samples of binary images of objects.

For modeling object shapes we employ moment descriptors to quantitatively capture the principal shape information of objects. To this end, image binarization is carried out on all images. Samples of resultant images after binarization are shown in Figure 4. This process removes textural details but preserves the whole shape of objects. Therefore, only holistic representation of objects is taken into consideration. Note that, the binarization process is performed in order to provide global outline of objects. In essence, in this section, we are looking for computational evidence to support psychological preference for basic level categorization as the entry level. It has been proposed that low spatial frequency information which forms the global appearance of objects is perceived before fine properties [12]. Our results provide support by demonstrating that basic categorization is not relied on in local processing and by employing global information through a shape based approach we can still reach high distinction between broad categories defined at this level.

As we mentioned before, the proposed feature vectors are constructed by moment-based descriptors. To this end, we computed the first eight standardized moments as well as Zernike moments. The simplest moment computes the center of mass in both directions. The second moment measures the variation from the center of the object in vertical and horizontal directions. Skewness is the third moment which measures the orientation of a distribution in the *x* and *y* directions. We, therefore, used the absolute value of this parameter to treat equally the left and right skewness. Fourth moment is kurtosis and deals with the peakedness and tail weight of a distribution. The fifth to eighth moments quantify higher shape parameters. We further calculated Zernike moments to obtain richer shape characteristics of objects. Zernike moments are constructed by projection over a sequence of orthogonal basis polynomials [25] and they have shown to be effective in shape classification tasks [26, 27]. In our experiments, we used the magnitude of Zernike moments over 20 basis functions of order 6 (we used public codes released by Christian Wolf available at http://liris.cnrs.fr/christian.wolf). We then concatenated the feature vector obtained from standard moments with Zernike moments resulting in a 32-dimensional feature vector:

(7) bsFeat1:16=μ1,…,μ8,bsFeat16:32=Anm;n=6,  m=0,

where *μ*_*n*_ is a two-dimensional vector of the *n*th-order moment of the input image on both *x* and *y* directions and *A*_*nm*_ is the projection of the image into Zernike basis function of order *n* with repetition *m*.

### 4.2. Results and Discussion

To highlight the efficacy of this method, we compared our results with C2 features [22] and HOG descriptors [21] which are known as successful techniques for object recognition. In all cases, SVM classifiers with linear kernels are used. The C2 features are computed by HMAX model in a four-layered architecture (two S layers and two C layers which perform template matching and max-pooling, resp.). The final C2 features are the result of resemblance to the stored local patches (in our case 200 patches). Histogram of Gradients (HOG) descriptor is created by counting occurrence of different orientations inside grids and concatenating them into a vector. We applied the basic form of HOG algorithm by dividing each image into 4 by 4 nonoverlapping blocks and calculating orientation histograms with 8 bins over each block. Thereupon, each input image is described with 200 dimensions using HMAX model and with 128 dimensions using HOG method. In contrast, in the proposed moment-based approach, each image is represented with an input vector of length 32. Nevertheless, it can be understood from Table 4 that the proposed global approach achieves better performance compared to the other local powerful methods (higher total accuracy and lower time complexity in comparison to C2 features). It is remarkable that while the set of statistical moments are simple, computationally cheap, and fast to be processed on both training and test phases, they attain high performance. It can be argued that shape-based approaches are preferable in situations when high resolution images are not available or cannot be stored due to memory space issues and information bottlenecks. This may not seem to be a serious problem regarding the tremendous development in memory technologies. However, it is highly profitable and biologically arguable to take an approach which is not dependent on consuming large volume of memory. The results also suggest that shape properties are a rich source of information for classification of general categories of animal and plant. One explaining factor is high degree of feature sharedness among members of general concepts [28] which boosts the structural similarity within each group. Studies towards global representation of objects are also important for mind and brain research. For instance, it has been shown that patients with semantic impairment have difficulties to access subordinate knowledge [29] but yet not much is known about the characteristics of the type of knowledge and its internal representation in brain. The lateral occipital complex (LOC) in human brain has been found to be involved in visual shape processing of objects [30]. In particular, it has been shown that LOC activation is related to shape characteristic of objects rather than specific features such as edge [31]. More studies and experiments are required to be conducted in order to probe the interplay between low level visual area (e.g., V1) and higher level visual area (e.g., LOC) as well as the underlying visual mechanism regarding to relationship between local and global visual processing.

## 5. Subordinate Level of Recognition

While the categories associated to the superordinate and basic concepts are demonstrated to be well distinguishable by utilizing global features (Sections 3 and 4), detailed information is required in order to capture fine distinction within subordinate categories. For example, while quadruped animals such as cougar and elephant can be distinguished from flowers such as sunflower and water lily by using global shape information, further local processing is required to tell them apart. Initial support for this argument comes from biological studies about coarse-to-fine processing in visual system analysis [32, 33] or global-to-local approaches [34–36]. In this section, we investigate whether visual characteristics collected from conceptual space encompass efficient information for recognition of subcategory objects. In other words, we question whether it would be beneficial to define feature vectors for subcategories of animal and plant by driving specific information about each conceptual space. In essence, we propose an approach that utilizes conceptual space information for feature extraction. For this purpose, we divide natural superordinate category into two subcategories of animal and plant on training samples. Then we develop local features based on information extracted from each subspace.

### 5.1. Method

Our strategies are developed based on the idea that conceptual knowledge can provide detailed information about structure of each basic class. To peruse this idea, we take an approach similar to prototype matching in which we use PCA method. All images are first cropped to eliminate border area and then rescaled to 100 × 100 pixels. Next, the eigenvectors of covariance matrix of all training images are generated. Note that, instead of generating the covariance matrix of the stimuli set (i.e., *SS* ^*T*^), which is a very large (*N* ^2^ × *N* ^2^, *N* = 100) dimensional matrix, we compute the covariance matrix associated to the transpose of *S* (i.e., *S* ^*T*^ *S*) [37]. Thus, the relationship between the eigenvectors of covariance matrix of *S* (i.e., *u*_*i*_) and the eigenvectors of the covariance of *S* ^*T*^ (i.e., *v*_*i*_) can be expressed by

(8) $$\begin{array}{c} u_{i}=Sv_{i}. \end{array}$$

Feature vectors corresponding to each image are generated by projection of image's pixels on the computed eigenvectors. We create eigenvectors in two modes: (1) flat mode, in which eigenvectors are calculated over all images of animals and plants (flat space), and (2) conceptual mode, in which eigenvectors are calculated on each subspace of animal and plant separately and then the results are concatenated. In other words, in flat recognition, feature representation for images is made by projection on the eigenvectors derived from the flat space, whereas, in the conceptual recognition, the feature representation of each image is built by concatenating the projection on both subspaces. The following describes subcategory feature creation:

(9) $$\begin{array}{c} subFeat\left(\;S\;\right)=\left\{\begin{array}{cc} u_{AP}^{T}S & flat\;\;mode\\ \left[u_{A}^{T}S,u_{P}^{T}S\right] & conceptual\;\;mode, \end{array}\right. \end{array}$$

where *u*_*AP*_ denotes the eigenvectors obtained from training samples including both animal and plant categories, while *u*_*A*_ and *u*_*P*_ represent eigenvectors computed over animal samples and plant samples, respectively.

**Figure 5:**
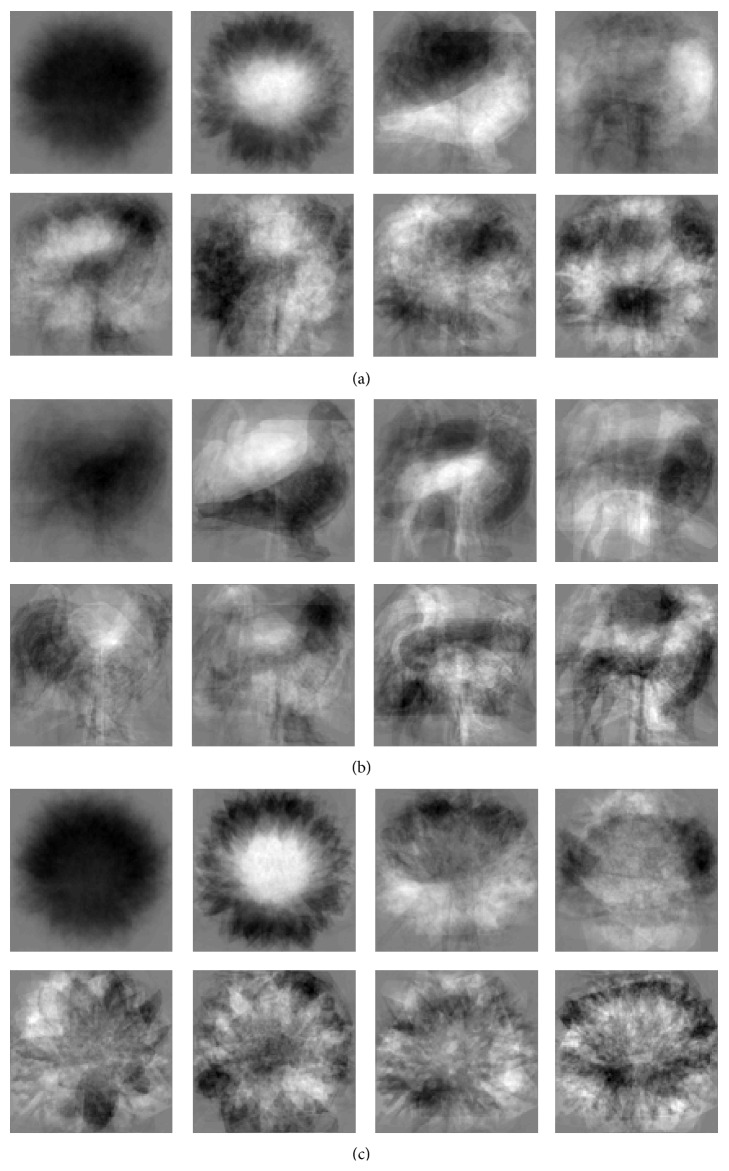
Eigen matrices associated to (a) flat space, (b) conceptual animal subspace, and (c) conceptual plant subspace.

Figure 5 demonstrates the first eight eigenvectors associated to each conceptual subspace using dataset 1. It can be inferred that eigenvectors associated to the flat space are not as informative as eigenvectors derived from conceptual space.

### 5.2. Results and Discussion

**Figure 6:**
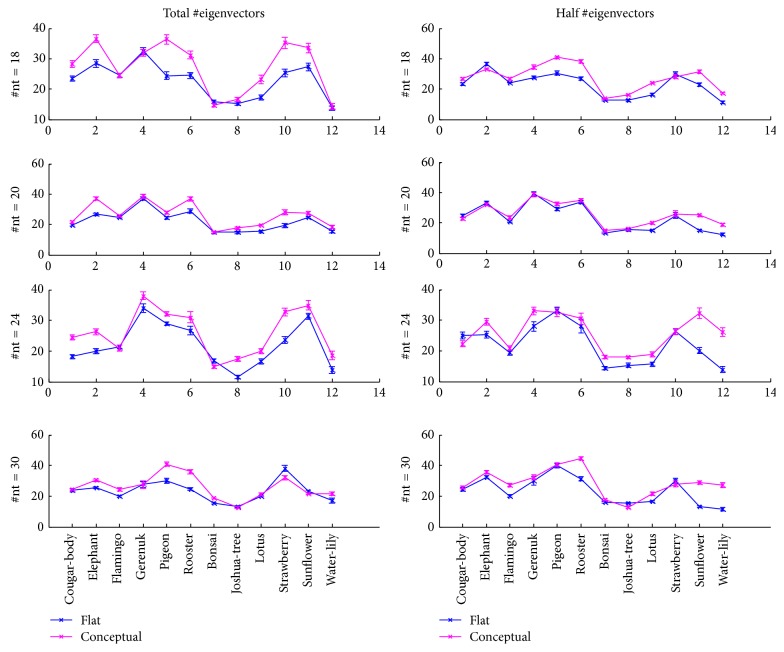
Categorization accuracy (nt: number of training samples). Total number of eigenvectors are equal to the total number of training samples.

**Figure 7:**
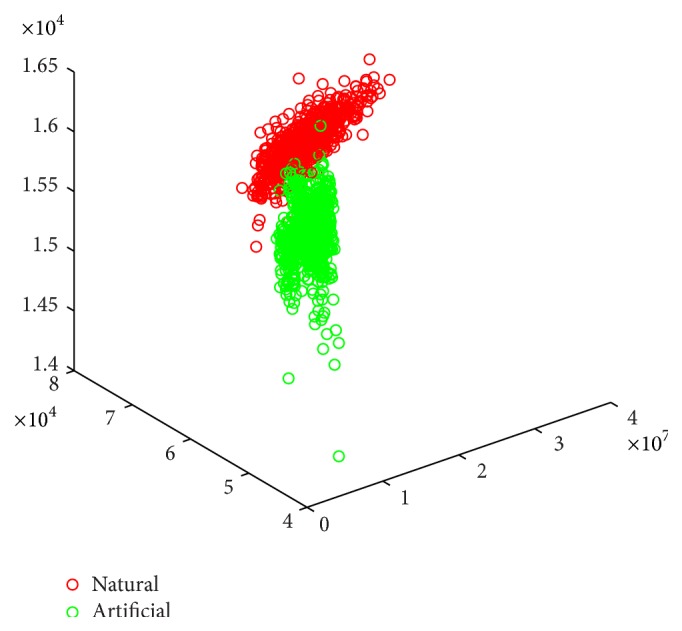
Scatter plot for all data represented by frequency features. Artificial and natural images are represented with green and red circles correspondingly.

**Figure 8:**
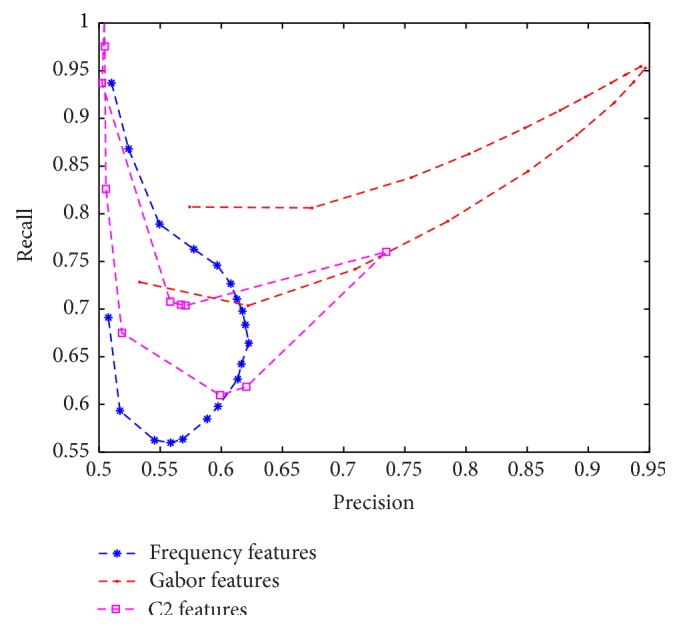
Precision-recall curve. The results are obtained by using different threshold values on the result of fuzzy clustering.

**Figure 9:**
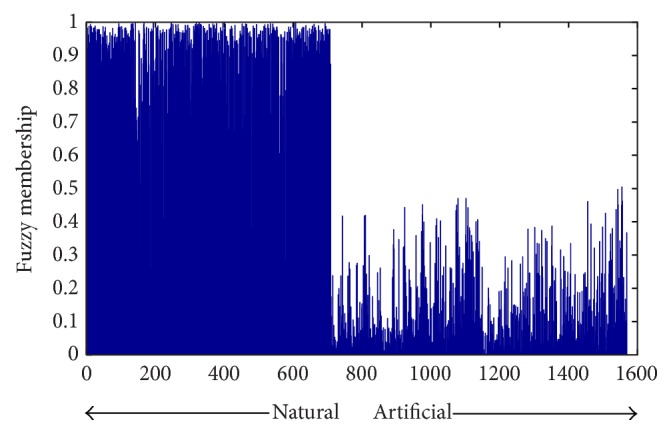
Fuzzy membership grade. Each bar shows the degree membership of each data to natural fuzzy cluster.

For evaluation, we compared accuracy of flat recognition to that of conceptual recognition. In our method, different random sets of train and test samples are created at each iteration and the results are averaged over ten independent runs. In addition, we use SVM classifier with linear kernel for evaluation. The performance associated to subordinate categories of dataset 1 in terms of their mean and standard deviation of the mean is illustrated in Figure 6. The number of training samples used in each experiment is indicated by nt variable. Note that total number of eigenvectors is equal to the number of training images used in each subspace. This is denoted by* total* eigenvectors. It can be implied that the classification performance has been improved in almost all classes. We also evaluated the percentage improvements through all categories. To this end, the number of training samples is changed between 50% and 75% of whole data and the feature dimensions are tested with both total number of obtained eigenvectors and half of them. The best obtained performance of the average percentage improvement over all classes is 22.12% for dataset 1 and 30.26% for dataset 2. These results suggest that deploying knowledge of conceptual categories of animal and plant improves the accuracy of subcategory recognition. These results are achieved due to high intraclass similarity of basic groups and their low interclass similarity. The abstract information that accounted for the main characteristic of features of each conceptual space is captured by the aid of eigenvectors derived from covariance matrix corresponding to each space. Therefore, projection of images on both conceptual spaces concretely measures the similarity proportion of each space. The weight vectors obtained after image projection are then served as an initial prediction of subcategory candidates. The ultimate decision is made by a classification task that utilizes a vector of predictions. In contrast, in the flat mode, where no specific conceptual knowledge is provided, eigenvectors carry combined information from both spaces and hence they fail to attain fine subcategory distinctions. Note that in this section we showed only one possible implementation that reveals the impact of conceptual knowledge on boosting the object recognition rate. Our future work will concentrate on developing more powerful methods that benefit from taxonomic knowledge.

## 6. Conclusion

In this study, we investigated visual representation of concepts at three levels of inclusiveness. The concepts of each level are known as superordinate, basic, and subordinate categories. To make distinction between superordinate categories at the first level (i.e., artificial and natural concepts), we used energy of frequency spectrum of images and showed its superiority compared to two other methods. For basic category representation in the second level (i.e., animal and plant concepts), we proposed to utilize moment descriptors in order to capture the differences in shape rather than local patches of images. The results demonstrated overall better performance to that of local based methods. Finally, we showed that space decomposition based on conceptual categories can be beneficial in terms of accuracy in recognition of subordinate object classes. Our attempt in all the three phases was motivated from cognitive theories to delineate a consistent computational model. The superordinate and subordinate categories both stand at a lower level of cue validity than basic level which indicates the basic category is the most inclusive level [8]. Accordingly, in our approach, the first and third levels of recognition rely on gray scale information, but the second level of recognition is based on shape properties obtained through processing of binary images.

## Figures and Tables

**Table 1:** Levels of abstraction.

Level of taxonomy	Example
The superordinate level	Animal
The basic level	Dog
The subordinate level	Reriever

**Table 2:** Object categories in taxonomic structure.

	Dataset 1			Dataset 2			Dataset 3	
Superordinate level	Natural		Artificial	Natural		Artificial	Natural	
Basic level	Animal	Plant		Animal	Plant		Animal	Plant

Subordinate level	Flamingo, pigeon, rooster, cougar-body, elephant, gerenuk	Sunflower, water-lily, lotus, strawberry, bonsai, Joshua-tree	Obj1, obj3, obj5, obj6, obj7, obj8, obj9, obj10, obj11, obj12, obj14, obj15	Bird, cat, dog	Bonsai, greenplant, tree	Balloon, bucket, cooler, horseshoe, mattress	Bat, bird, chicken, deer, elephant, horse	Apple, Device 0 (flower 0), Device 1 (flower 1), Device 2 (flower 2), Device 7 (flower 3), tree

**Table 3:** Clustering evaluation results.

	Dataset 1				Dataset 2			
	*F*-measure	Precision	Accuracy	Recall	*F*-measure	Precision	Accuracy	Recall
Frequency features	**94.96**	**93.78**	**95.00**	**94.30**	**65.91**	**62.88**	**62.12**	**66.13**
Gabor feature	71.80	72.34	62.56	72.32	62.60	62.30	62.06	62.61
C2 features	75.91	84.33	74.07	76.01	51.27	51.54	51.27	51.01

**Table 4:** Comparison results of classification on basic conceptual categories. Results are averaged over 10 iterations. Time complexity is averaged over all train samples.

Features	Dataset 1				Dataset 3				#Feature vector dimensions
	Accuracy			Average processing time per sample	Accuracy			Average processing time per sample	
	Animal class	Plant class	Total (over all test samples)		Animal class	Plant class	Total (over all test samples)		
C2	86.55 (2.66)	84.33 (2.98)	85.27 (2.41)	3.56	93.50 (2.28)	**95.50** (3.14)	94.50 (1.48)	7.23	200

HOG	82.37 (3.21)	80.33 (1.62)	81.13 (1.57)	**0.0385**	85.66 (4.02)	94.66 (2.58)	90.16 (2.03)	**0.1452**	128

Moment-based method	**86.67** (2.57)	**85.35** (2.78)	**85.71** (.79)	0.1465	**94.66** (3.16)	95.16 (1.99)	**94.92** (1.93)	0.2902	**32**

## Appendix

The corresponding scatter plot associated to the two first dimensions of frequency features on dataset 1 are plotted in Figure 7. Natural and artificial objects are denoted by red circles and green dots consecutively. This figure indicates that features defined at the superordinate level map data into a space where they are linearly separable. The precision-recall curve that is obtained by using different threshold values on the result of fuzzy c-means clustering is shown in Figure 8. Besides, fuzzy memberships for all artificial and natural test data are illustrated in Figure 9. The blue bars indicate the degree of naturalness.
